# Gene Expression Signatures of Immaturity, Decreased pH, and Neural Hyperexcitation in the Hippocampus of Alzheimer's Disease Model Mice

**DOI:** 10.1002/npr2.70001

**Published:** 2025-02-05

**Authors:** Sayaka Naganishi, Hideo Hagihara, Tsuyoshi Miyakawa

**Affiliations:** ^1^ Department of Systems Medical Science Fujita Health University Graduate School of Medicine Toyoake Aichi Japan; ^2^ Division of Systems Medical Science, Center for Medical Science Fujita Health University Toyoake Aichi Japan

**Keywords:** Alzheimer's disease, hyperexcitation, immaturity, pH, transcriptome

## Abstract

**Aims:**

Alzheimer's disease (AD) is a leading cause of dementia, with increasing prevalence. Mutations in genes like MAPT, PSEN1, and PSEN2 are risk factors, leading to the development of several AD model mice. Recent hypotheses suggest AD brain pathology involves abnormal neurodevelopment, decreased pH, and neural hyperexcitation. However, it remains unclear to what extent these pathologies are reflected in the gene expression changes of AD models. This study aims to compare gene expression patterns in the brains of multiple AD model mice with those related to these three factors, evaluating the extent of overlap.

**Methods:**

We conducted a comprehensive search of public databases, collecting 20 gene expression datasets from the hippocampus of AD model mice. These datasets were compared with gene sets related to hippocampal maturation, brain pH, and neural hyperexcitation to statistically assess overlap. Pathway enrichment analysis explored the biological relevance of these gene expression changes.

**Results:**

The extent of overlap with maturity‐, pH‐, and hyperexcitation‐associated genes varied across AD models, showing significant correlations between lower maturity, lower pH, and increased neural hyperexcitation. In MAPT mutant and APP+PSEN1 homozygous transgenic mice, these signatures became more pronounced with age. Pathway meta‐analysis revealed that genes associated with maturity, pH, and hyperexcitation in AD models are involved in synaptic and channel functions, as well as inflammatory responses, consistent with previous studies.

**Conclusion:**

These findings suggest that pathophysiological changes related to maturity, pH, and neural hyperexcitation play varying roles across individual AD model mice. Our recent study found a negative correlation between disease progression and actual pH levels in human AD patients. Considering the results presented in this study, maturity and neural hyperexcitation, which are correlated with pH, may also be linked to disease progression. Thus, gene expression changes in these factors could be useful markers for assessing the pathology in AD models.

## Abstract 2

Analyses of the 20 gene expression datasets of hippocampus of Alzheimer's disease model mice revealed patterns linked to immaturity, decreased pH, and neural hyperexcitation. Pathway analysis suggests that the gene expression changes might be involved in synaptic and channel functions, as well as inflammatory responses.

## Graphical abstract

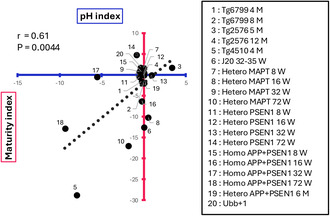

## 1 Introduction

Alzheimer's disease (AD) is one of the most common causes of dementia, and the number of patients has been increasing in recent years [1]. Thus far, the amyloid precursor protein (APP) gene, as well as the presenilin 1 (PSEN1), presenilin 2 (PSEN2), and microtubule‐associated protein tau (MAPT) genes, have been identified as disease‐associated genes (Alzforum, http://www.alzforum.org) [2, 3, 4, 5]. These discoveries have led to the development of various transgenic mice, primarily overexpressing APP or APP/PSEN1. These model mice have been widely utilized as essential research tools for understanding the mechanisms underlying AD [3, 6, 7]. The most prominent pathological features of AD include neuronal cell death due to the accumulation of amyloid β (Aβ) and the aggregation of hyperphosphorylated tau [8], as well as brain atrophy, all of which have been linked to cognitive decline [9, 10]. Accumulating evidence has led to various discussions and considerations regarding other aspects of AD pathology, among which we have particularly focused on the following three hypotheses. First, a phenomenon, “immature Dentate Gyrus (iDG),” in which the molecular expression patterns and electrophysiological properties in the DG of adult mice resemble those of typically developing infants or adolescents, has been observed in various neuropsychiatric disorders, including schizophrenia/intellectual disability, bipolar disorder, epilepsy, and AD, in humans and model mice [11, 12, 13, 14, 15, 16, 17, 18, 19]. Notably, the iDG phenotype can be induced by chronic antidepressant administration and experimental neural hyperexcitation in pre‐existing mature DG neurons [20, 21, 22, 23, 24, 25]. We refer to the phenomenon in which mature neurons reversed to a pseudoimmature state as “dematuration” [20, 25]. There are reports of decreased adult neurogenesis [26, 27, 28, 29], while other studies indicate an increase in AD [30, 31, 32, 33], both in humans and in model mice. Findings on increased neurogenesis are often the result of using doublecortin, an immature neuronal marker. However, as the marker can be re‐expressed through the process of dematuration, careful attention should be paid when interpreting studies on adult neurogenesis [25]. Second, pH measurements conducted using postmortem brain samples and magnetic resonance spectroscopy (MRS) have revealed that decreased pH levels in patients with AD, as well as in several other neuropsychiatric disorders, and their corresponding animal models [34, 35, 36, 37, 38, 39]. We also found through gene expression pattern analyses that expression changes correlated with pH reduction occur in the postmortem brains of AD patients [40]. Third, neuronal hyperactivity has been implicated in AD in both humans and mouse models [41]. Epileptic‐like spikes have been observed in the brains of AD patients, and spontaneous seizures can occur in AD model mice [42, 43, 44]. Importantly, Aβ and tau are suggested to promote neural hyperexcitability [45, 46], and seizures are thought to enhance Aβ and tau deposition, thereby accelerating neurodegenerative processes [47]. These findings suggest a bidirectional relationship between Aβ and tau deposition and neural hyperexcitation [48].

In our previous study, we evaluated AD model mouse data from the perspective of gene expression patterns associated with immaturity and neural hyperexcitation [49]. However, the previous study was based on a small number of datasets in AD models. Thus, we need to conduct a comprehensive analysis of datasets in AD model mice. The pathogenesis of AD is thought to result from the interaction of various biological and environmental factors [50]. Therefore, understanding the relationships between abnormalities in maturity, pH changes, and neural excitation is crucial for gaining deeper insights into the pathophysiology of AD. It remains unclear to what extent these three signatures associated with AD contribute to gene expression changes in each mouse model. In this study, we comprehensively collected publicly available datasets from BaseSpace [51], a database containing over 260 000 gene expression datasets. We then compared the patterns of gene expression changes in AD model mice with three gene expression signatures: hippocampal maturation, brain pH changes, and neural hyperexcitation. While the types of samples used were diverse, gene expression changes associated with these three signatures in AD model mice were found to be statistically significant. Obtaining statistically significant results from heterogeneous data, which are generally hard to compare, was made possible by applying the bioinformatics method called the Running Fisher algorithm [51]. By adopting a ranking approach based on normalized fold‐change, it becomes possible to compare data across different studies, species, and analysis platforms [51]. We also conducted pathway analysis to gain insights into the biological characteristics of gene expression associated with these three signatures in AD model mice. The analysis reveals that gene expression associated with hippocampal maturation and pH changes in AD pathology linked to synapse and channel activity‐related genes, while those associated with neural hyperexcitation involved in inflammatory responses‐related genes.

## 2 Materials and Methods

All data used in this study were publicly available gene expression data. A summary of the datasets, including the study name, number of samples, and age, is presented in Table S1.

### 2.1 Datasets of Alzheimer's Disease Model Mouse

We comprehensively queried gene expression datasets of AD model mice from the BaseSpace database using “Alzheimer's disease” and “hippocampus” as keywords (Table 1). In addition, we obtained a dataset of mice harboring the Sweden, Indiana mutation in the APP gene (J20 line) was obtained from the Gene Expression Omnibus of National Center for Biotechnology Information (GSE14522) [52], and manually imported the list of differentially expressed genes (DEGs) into BaseSpace following the manufacturer's instructions. DEGs were calculated between APP transgenic mice injected with a lentivirus expressing GFP and non‐transgenic mice with the same lentivirus, using a criterion of an absolute fold change > 1.2 and a *t*‐test *p*‐value < 0.05, with GEO2R provided on the platform. In total, 20 AD model datasets were analyzed in this study (Table 1 and Table S1).

**TABLE 1:** Summary of data used in this study.

Dataset name	Mutation	DEGs	Reference	GSE
Tg6799 4 M	APP mutation (Sweden, Florida, London mutation) + PSEN1 mutation	508	[53]	GSE52022
Tg6799 8 M	APP mutation (Sweden, Florida, London mutation) + PSEN1 mutation	286	[53]	GSE52022
Tg2576 5 M	APP mutation (Sweden mutation)	423	[54]	GSE36237
Tg2576 12 M	APP mutation (Sweden mutation)	319	[55]	GSE1556
Tg4510 4 M	MAPT mutation	2478	[56]	GSE53480
J20 32‐35 W	APP mutation (Sweden, Indiana mutation)	540	[15]	GSE14522
Hetero MAPT 8 W	Heterozygous MAPT mutation 8 week	92	[57]	GSE64398
Hetero MAPT 16 W	Heterozygous MAPT mutation 16 week	479	[57]	GSE64398
Hetero MAPT 32 W	Heterozygous MAPT mutation 32 week	84	[57]	GSE64398
Hetero MAPT 72 W	Heterozygous MAPT mutation 72 week	802	[57]	GSE64398
Hetero PSEN1 8 W	Heterozygous PSEN1 mutation 8 week	92	[57]	GSE64398
Hetero PSEN1 16 W	Heterozygous PSEN1 mutation 16 week	76	[57]	GSE64398
Hetero PSEN1 32 W	Heterozygous PSEN1 mutation 32 week	118	[57]	GSE64398
Hetero PSEN1 72 W	Heterozygous PSEN1 mutation 72 week	288	[57]	GSE64398
Homo APP+PSEN1 8 W	Homozygous human APP and PSEN1 mutation 8 week	330	[57]	GSE64398
Homo APP+PSEN1 16 W	Homozygous human APP and PSEN1 mutation 16 week	766	[57]	GSE64398
Homo APP+PSEN1 32 W	Homozygous human APP and PSEN1 mutation 32 week	1065	[57]	GSE64398
Homo APP+PSEN1 72 W	Homozygous human APP and PSEN1 mutation 72 week	1389	[57]	GSE64398
Hetero APP+PSEN1 6 M	Heterozygous APP and PSEN1 mutation 6 month	24	[58]	GSE248245
UBB + 1	Overexpressing UBB + 1 (ubiquitin B mutation)	506	[59]	GSE13691

### 2.2 Maturity‐Associated Genes

We used our previously published gene expression datasets, which examined maturational changes in the DG of wild‐type mice, comparing adults (33 weeks old; GSE42778) with infants (postnatal days (PD) 8, 11, 14, 17, 21, 25, and 29; GSE113727). Based on prior analysis, we selected PD8 mice for the infant data among them [49] (see Table S2 for details). We denoted genes that showed significant changes in expression from PD8 to adulthood, with an absolute fold change > 1.2 and a *t*‐test *p*‐value < 0.05, as maturity‐associated genes.

### 2.3 pH‐Associated Genes

The pH‐associated genes were previously defined [40]. These genes were originally identified through a meta‐analysis of gene expression data from normal human postmortem cortex obtained from multiple studies [60]. In this study, we used genes with a meta‐analysis *Q*‐value < 0.001, including 39 pH‐upregulated genes whose expression levels were positively correlated with pH values, and 268 pH‐downregulated genes whose expression levels were negatively correlated with pH values (see Table S6 in Mistry and Pavlidis 2010 [60]). The list of these genes (gene name and the summary statistics *S*‐value calculated for each gene, which was used as fold‐change) was imported into BaseSpace.

### 2.4 Neural Hyperexcitation‐Associated Genes

We used a gene expression dataset from the DG of rats that experienced pilocarpine‐induced epileptic seizures (GSE47752) [61]. The expression levels of 7073 genes were significantly altered in the DG of rats 1  day after pilocarpine injection compared to saline‐treated rats (absolute fold change > 1.2, *p* < 0.05), and these genes were denoted as neural hyperexcitability‐associated genes.

### 2.5 Evaluation of Similarity of Gene Expression Patterns

To examine whether and to what extent gene expression patterns in AD model mice are related to those of maturity‐, pH‐ and neural hyperexcitation‐associated genes, we compared them using the Running Fisher algorithm in BaseSpace [51]. This algorithm is a non‐parametric rank‐based statistical method that takes into account rank information based on the absolute fold change and the direction of expression changes within each gene set [51]. The similarity of gene expression patterns between the two conditions was assessed by the calculated overlap *p*‐value; the higher the similarity, the lower the resulting overlap *p*‐value [62, 63, 64].

### 2.6 Maturity Index, pH Index, and Hyperexcitation Index

**FIGURE 1:**
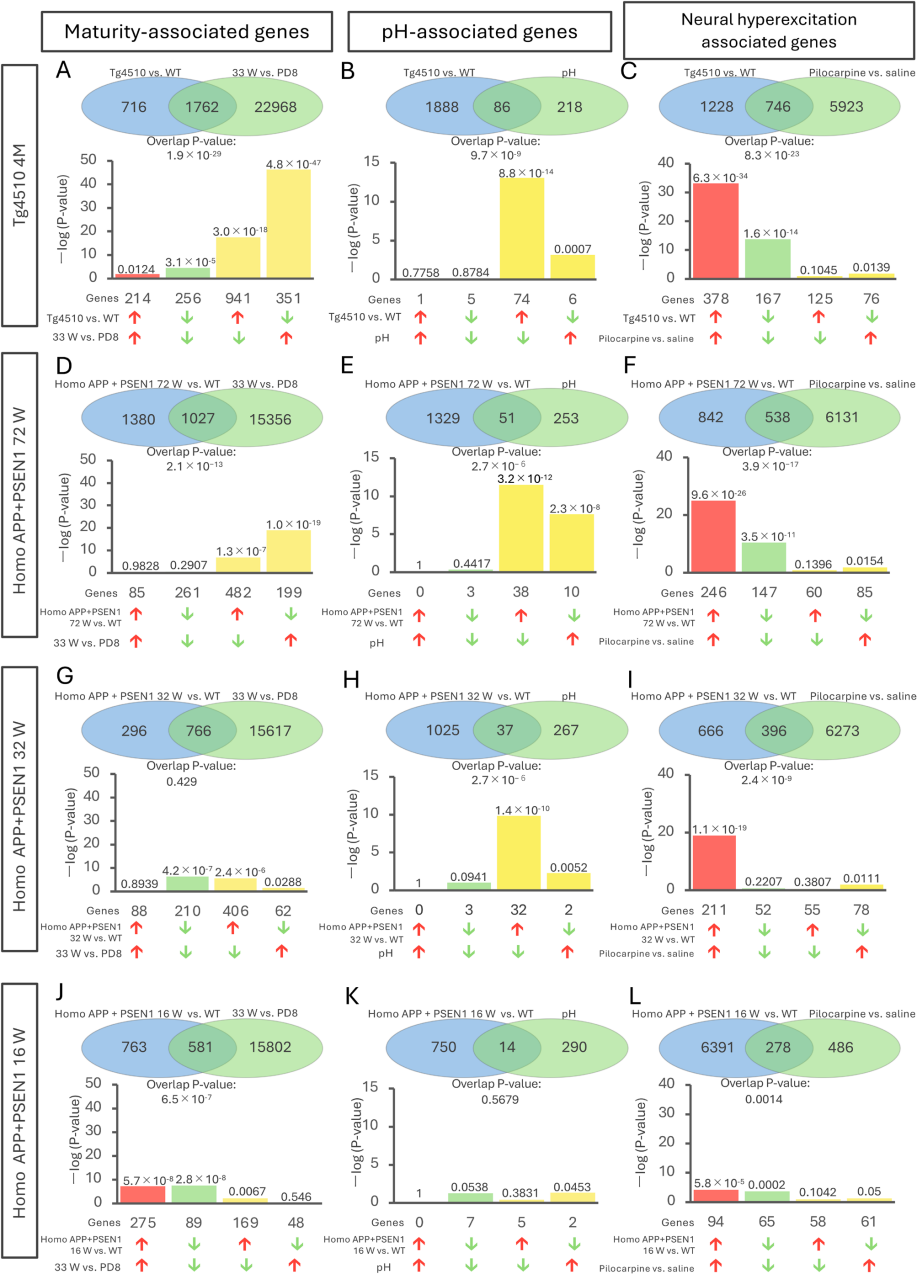
The extent of overlap with maturity‐, pH‐, and neural hyperexcitation‐associated genes varies across different AD model datasets. (A–C) Venn diagrams illustrating the comparison of differentially expressed genes (DEGs) of Tg4510 mice with maturity‐associated genes (A), pH‐associated genes (B), and neural hyperexcitation‐associated genes (C). Bar graphs illustrate the *p*‐values for the overlap of genes regulated in the same direction (upregulated: Red bars; downregulated: Green bars) under both conditions or in opposite directions (yellow bars) in each condition. (D–L) The same analyses were performed for different AD model datasets: Homo APP+PSEN1 72w mice (D–F), Homo APP+PSEN1 32w mice (G–I), and Homo APP+PSEN1 16w mice (J–L). Red and green arrows in AD model mice: Genes with increased (red) or decreased (green) expression in AD model mice compared to WT mice; Red and green arrows in 33 W vs. PD8: Genes with increased (red) or decreased (green) expression in 33 W compared to PD8; Red and green arrows in pH: Genes with expression positively (red) or negatively (green) correlated with pH; Red and green arrows in pilocarpine: Genes with increased (red) or decreased (green) expression in pilocarpine compared to saline. Note that the vertical axis scales are as follows: 0–50 for A, D, G, and J; 0–15 for B, E, H, and K; and 0–40 for C, F, I, and L. PD, postnatal days; W, weeks old.

The calculated overlap *p*‐values were transformed to obtain a similarity index, indicated by the maturity index, pH index, and hyperexcitation index, according to the following formula (Figure 1):

Maturity index = −log10 (overlap *p*‐value between maturity‐associated genes and DEGs in AD model mouse) (Figure 1B,D).

pH index = −log10 (overlap *p*‐value between pH‐associated genes and DEGs in AD model mouse) (Figure 1B,F).

Hyperexcitation index = −log10 (overlap *p*‐value between neural hyperexcitation‐associated genes and DEGs in AD model mouse) (Figure 1D,F).

We obtained the maturity, pH, hyperexcitation indices for each dataset of AD model mouse and plotted them in two‐dimensional (2‐D) space to examine whether the extent of overlap between DEGs of an AD model and each of maturity‐, pH‐, and neural hyperexcitation‐associated genes is correlated across AD model datasets (Figure 3A–C).

### 2.7 Pathway Enrichment Meta‐Analysis

The Pathways and biogroups enriched in the genes of interest were identified through a combination of rank‐based enrichment statistics and biomedical ontologies using BaseSpace [15, 40, 49]. We meta‐analyzed 20 gene sets identified 20 AD model datasets each for maturity‐, pH‐, and neural hyperexcitation‐associated genes (Figure 4). This analysis included pathways and biogroups from GO and canonical pathways from Broad MSigDB.

## 3 Results

The extent of overlap with maturity‐, pH‐, and neural hyperexcitation‐associated genes varies across different AD model datasets.

We investigated whether, and to what extent, the gene expression patterns in AD model mice resemble those of genes associated with maturity, pH, and neural hyperexcitation. To this end, we compared DEGs from each AD model dataset with maturity‐, pH‐, and neural hyperexcitation‐associated genes using the Running Fisher algorithm provided on the BaseSpace platform. Results from four of the 20 datasets, including two strains of model mice of different ages (4‐month‐old Tg4510 and 16‐, 32‐, and 72‐week‐old Homo APP+PSEN1), are shown in Figure 1. Out of 2478 genes altered in the dataset of Tg4510 mice (DEGs from Tg4510 mice compared to wild‐type mice), 1762 were shared with maturity‐associated genes (DEGs in 33‐week‐old adult mice compared to PD8 infant mice). The overlap *p*‐value between the two conditions was 1.9 × 10^−29^, indicating a statistically significant similarity in gene expression changes between them (Figure 1A). Among the 1762 shared genes, 214 were upregulated (*p* = 0.012) and 256 were downregulated (*p* = 3.1 × 10^−5^) in both conditions (Figure 1A). These 214 and 256 genes with the same direction of change in expression between the two conditions are denoted as showing a positive correlation. We also found that 941 genes were upregulated in Tg4510 mice and downregulated in maturity‐associated genes (*p* = 3.0 × 10^−18^), and 351 genes were downregulated in Tg4510 mice and upregulated in maturity‐associated genes (*p* = 4.8 × 10^−47^) (Figure 1A). The genes that showed opposite changes between the two conditions are denoted as showing a negative correlation. Tg4510 mice dominantly showed a negative correlation with maturity‐associated genes, suggesting a lower maturity, or an iDG phenotype, in this model.

Similar analyses were performed for the same Tg4510 mouse dataset with pH‐associated genes and neural hyperexcitation‐associated genes. Tg4510 mice dominantly showed a negative correlation with pH‐associated genes (Figure 1B) and a positive correlation with neural hyperexcitation‐associated genes (Figure 1C), respectively, suggesting decreased brain pH and increased neural hyperexcitation in this model.

Together with the results from analyses of the other three datasets (Homo APP+PSEN1 mice with different age stages; Figure 1D–L), we found that the similarity, as assessed by overlap *p*‐values, and the predominant direction of correlation (positive or negative) with maturity‐, pH‐, and neural hyperexcitation‐associated genes varied depending on the dataset. Notably, even within the same mouse strain, the degree of overlap differed by age.

Lower maturity, lower pH, and increased neural hyperexcitation are correlated each other across AD mouse models in terms of gene expression patterns.

**FIGURE 2:**
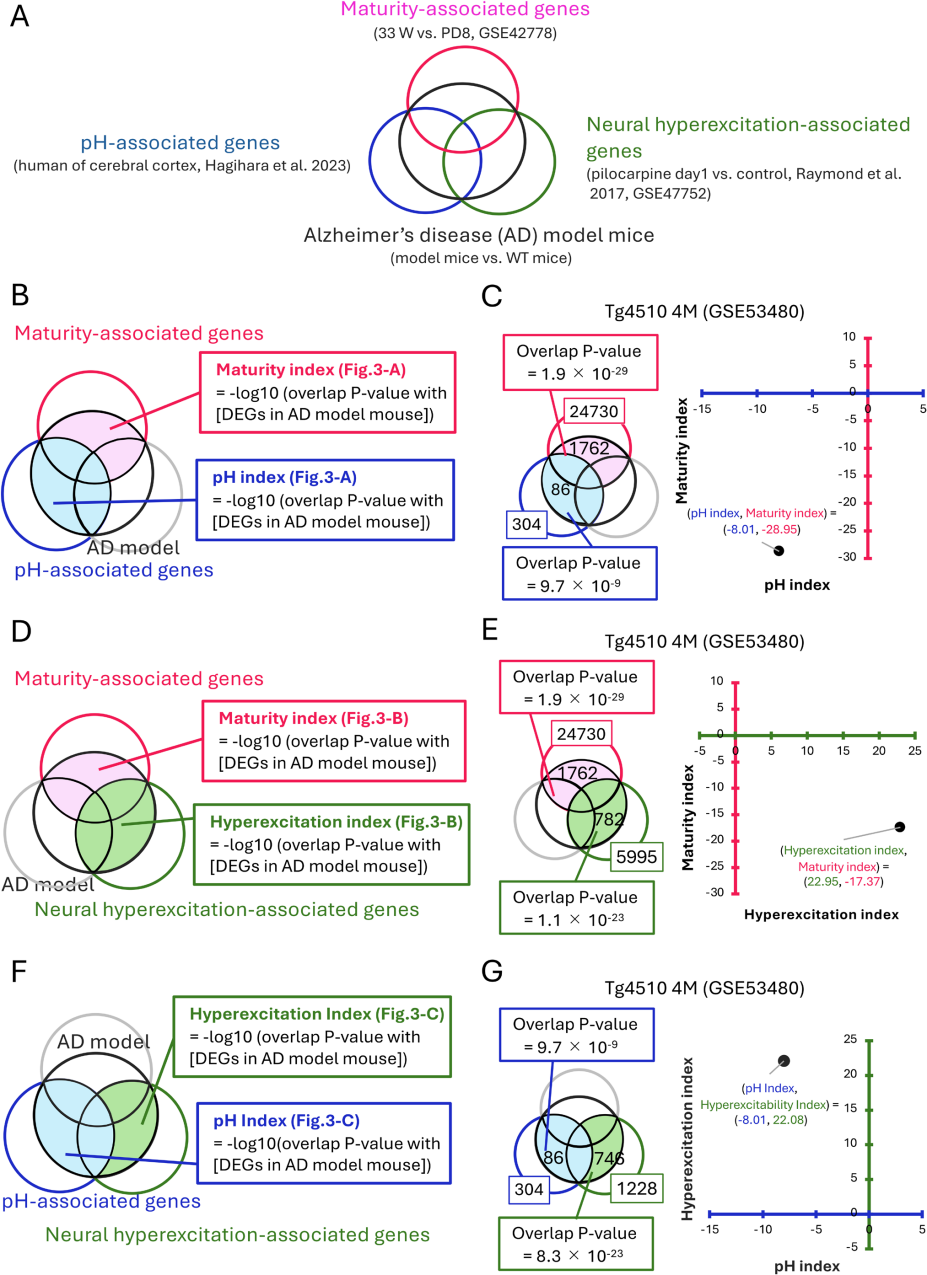
Overview of the two‐dimensional (2‐D) analysis of gene expression datasets in AD model mice. (A) Venn diagram illustrating the comparison between DEGs of AD models and maturity‐, pH‐, and neural hyperexcitation‐associated genes. The red circle represents maturity‐associated genes, the blue circle represents pH‐associated genes, the green circle represents neural hyperexcitation‐associated genes, and the black circle represents differentially expressed genes (DEGs) in the hippocampus of 20 different AD mouse models. (B) Calculation of the maturity index and pH index. Genes in the areas highlighted in pink and blue were used for the calculation of the maturity index and pH index, respectively. (C) 2‐D analysis based on the maturity index and pH index applied to a dataset of Tg4510 mice. There are 1762 common genes between the DEGs of Tg4510. (B) Mice and the maturity‐associated genes (highlighted in pink), and 86 common genes with the pH‐associated genes (highlighted in light blue), with overlap *p*‐values of 1.9 × 10^−29^ and 9.7 × 10^−9^, respectively. The maturity index and pH index for the Tg4510 dataset are therefore −8.01 and −28.95. This dataset is plotted on two‐dimensional coordinates defined by the maturity index (vertical axis) and pH index (horizontal axis). (D–G) Following the same procedure as in panels B and C, the same Tg4510 mouse dataset is analyzed based on the maturity index and neural hyperexcitation index (D, E) and the pH index and neural hyperexcitation index (F, G).

**FIGURE 3:**
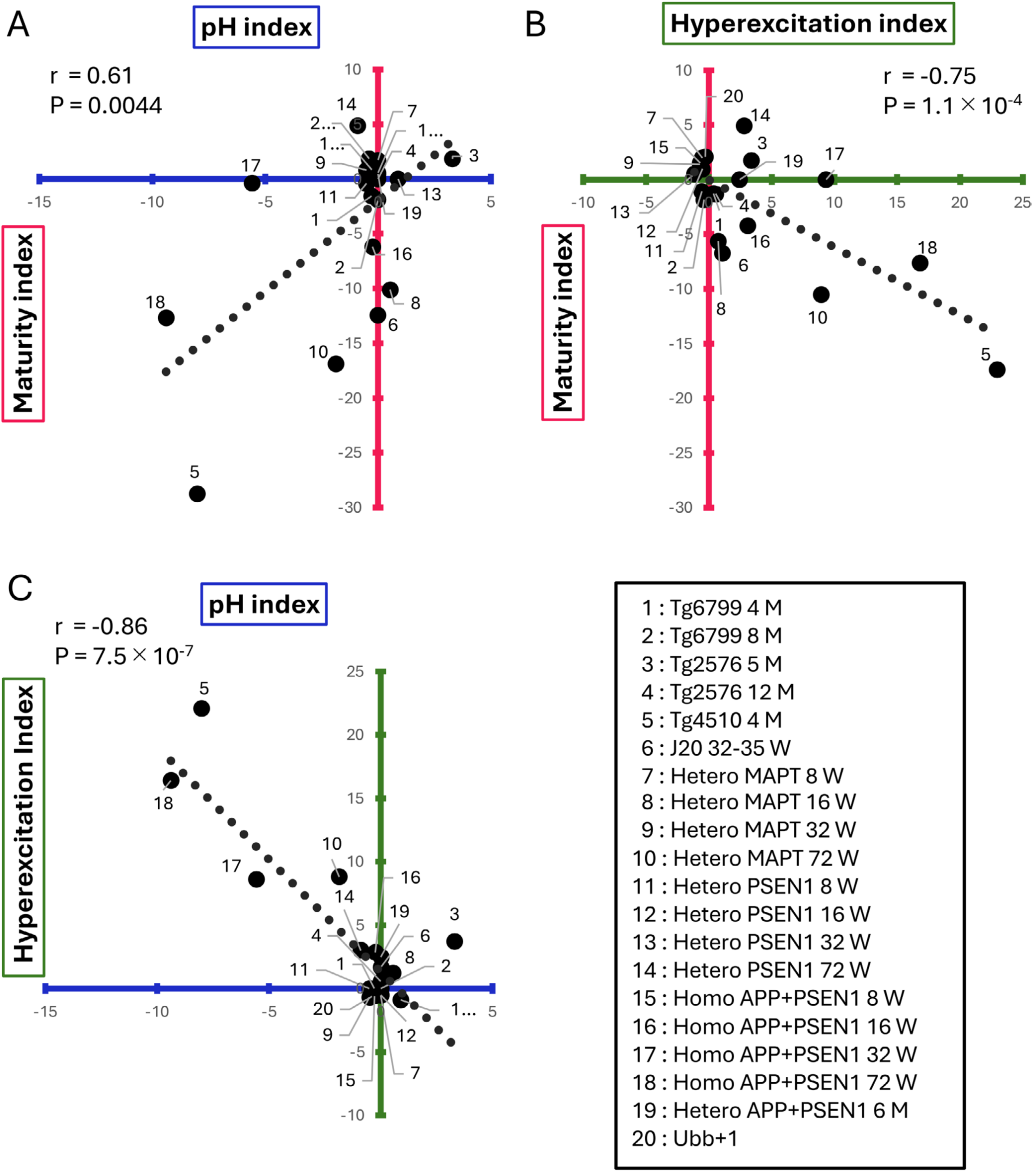
The extent of gene expression signatures related to immaturity, lower pH, and neural hyperexcitation are correlated across AD mouse models. (A) The scatter plot shows a positive correlation between the maturity index and pH index across 20 different AD model datasets. (B) The scatter plot shows a negative correlation between the maturity index and neural hyperexcitation index. (C) The scatter plot shows a negative correlation between the hyperexcitation index and pH index. The dotted lines represent the linear regression of the 20 data points.

Next, we investigated the extent to which the gene expression changes related to maturity, pH, and neural hyperexcitation overlap with the gene expression patterns in various AD model mice. As described above, we evaluated the similarities between changes in gene expression patterns across different AD model mice using overlap *p*‐values calculated with the Running Fisher algorithm. In this case, to investigate the correlations among the three indicators, we ensured that there was no overlap between the genes associated with each of them. Similarity indices for each comparison were defined as the −log of the overlap *p*‐values with maturity‐, pH‐, and neural hyperexcitation‐associated genes (Figure 2B,D,F). These are referred to as the maturity index, pH index, and hyperexcitation index, respectively. Higher values of the maturity, pH, and hyperexcitation indices indicate a greater overlap between the analyzed dataset and the maturity‐, pH‐, and neural hyperexcitation‐associated genes. We calculated the maturity, pH, and hyperexcitation indices for each dataset from AD model mice and plotted them in 2‐D space to illustrate the extent of overlap between the datasets and the maturity‐, pH‐, and neural hyperexcitation‐associated genes. We initially performed this 2‐D analysis on a dataset containing the maturity and pH indices using data from Tg4510 mice (Figure 2C). The number of genes showing expression changes in both Tg4510 mice and maturity‐ and pH‐associated genes were 1762 and 86, respectively (overlap *p*‐values = 1.9 × 10^−29^ and 9.7 × 10^−9^) (Figure 2C). Maturity and pH indices for this data were − 28.95 (= −log (1.9 × 10^−29^)) and −8.01 (= −log (9.7 × 10^−9^)). We applied the same procedure using the combinations of maturity and hyperexcitation indices, as well as hyperexcitation and pH indices (Figure 2E,G). The results of applying the procedure from Figure 2C,E,G to various AD model mice are shown in Figure 3.

As a result, a positive correlation was observed between the maturity and pH indices, a negative correlation between the maturity and hyperexcitation indices, and a negative correlation between the hyperexcitation and pH indices. Although the degree of these correlations varied among the AD model mice, all were statistically significant. Particularly in mice with Hetero MAPT or Homo APP+PSEN1 mutations, as the mice age, there is a stronger tendency for reduced maturity, decreased pH, and increased neural excitation. These findings suggest that the AD model mice exhibiting gene expression patterns associated with low pH tend to show increased neural hyperexcitation and lower maturity levels.

Maturity‐, pH‐, and neural hyperexcitation‐associated genes altered in AD model mice exhibit different biological properties.

**FIGURE 4:**
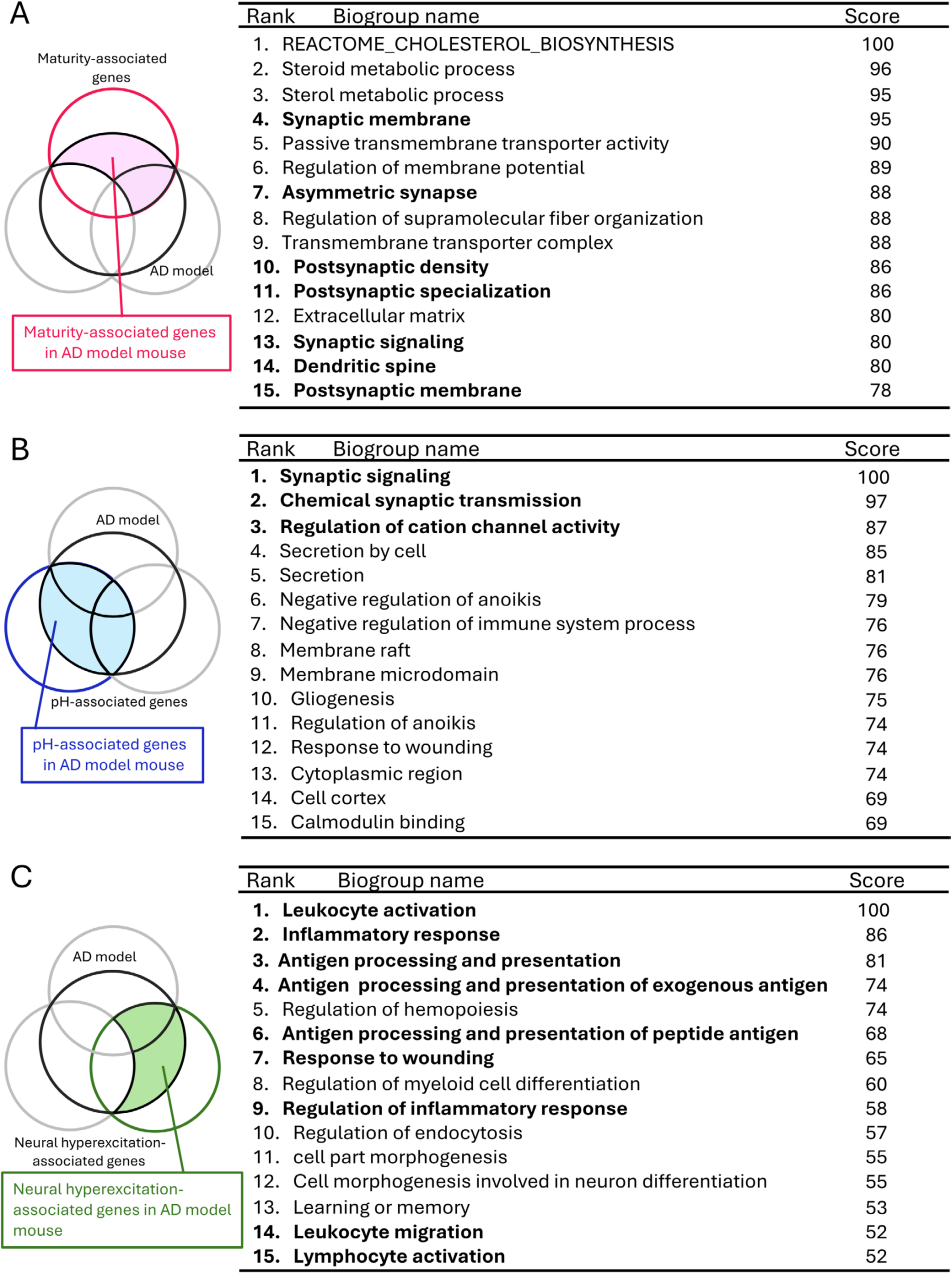
Pathway analysis of genes associated with maturity, pH, and neural hyperexcitation across 20 different AD model datasets. (A–C) Meta‐analysis of 20 gene sets showing overlapping features between each of the 20 AD model datasets and maturity‐associated genes (area filled in pink; A), pH‐associated genes (area filled in blue; B), and neural hyperexcitation‐associated genes (area filled in green; C). The top 15 pathways/biogroups are shown.

To characterize the biological features of maturity‐, pH‐, neural hyperexcitation‐associated genes altered in AD model mice, we conducted pathway enrichment analyses in BaseSpace. Among the maturity‐associated genes in AD model mouse, 7 out of the top 15 biogroups are associated with synapse (synaptic membrane and synaptic signaling; Figure 4A). Among the pH‐associated genes in AD model mice, 3 of the top 15 biogroups are associated with synapse and channel activity, including synaptic signaling, chemical synaptic transmission, and regulation of cation channel activity (Figure 4B). Among the neural hyperexcitation‐associated genes in AD model mouse, 9 out of the top 15 biogroups are associated with synapse (leukocyte activation, inflammatory response; Figure 4C). These results suggest that the maturity‐, pH‐ and neural hyperexcitation‐associated genes may be involved in different aspects of biological process in the brain of AD model mice.

## 4 Discussion

This study revealed that gene expression signatures related to maturity, pH, and neural hyperexcitation were found in the hippocampus of several AD model mice. Pathway analysis suggested that maturity‐ and pH‐associated genes in AD models are involved in synapse and channel activity, while neural hyperexcitation‐associated genes in inflammatory response. This study revealed that gene expression signatures related to maturity, pH, and neural hyperexcitation were present in the hippocampus of several AD model mice. Pathway analysis suggested that maturity‐ and pH‐associated genes in AD models are involved in synaptic and channel functions, while neural hyperexcitation‐associated genes are linked to inflammatory responses.

The extent of overlap with three signatures gene varies across different AD mouse models. The Tg4510 and J20 mice are examples of model organisms that exhibit more pronounced gene expression changes associated with these three signatures than other AD model mice. These two strains of mouse models have been reported to show a significant reduction in hippocampal volume compared to non‐transgenic mice [65, 66]. On the other hand, Tg6799 mice, and Tg2576 mice, which exhibit less gene expression changes related to the three signatures, do not show a significant reduction in hippocampal volume [67, 68]. These findings suggest that the variation in the levels of changes in the three signatures may be associated with hippocampal volume. Our recent study found a negative correlation between AD progression, stratified by Braak stage, and actual pH levels in postmortem brains of human AD patients [38]. Considering this, maturity‐ and neural hyperexcitation‐associated genes, which are correlated with pH‐associated genes, may also be correlated with AD progression. In this study, it was confirmed that in MAPT mutant mice and Homo APP+PSEN1 mutant mice, these signatures became more pronounced with increasing age. Therefore, this suggests that maturity, pH, and neural hyperexcitation are key factors in the pathogenesis of AD.

Pathway analysis indicated that genes related to maturity‐ and pH‐associated genes in AD models were linked to synaptic and channel functions. Our previous studies showed that hyperexcitation‐induced maturity marker genes (hiM genes) and genes with increased expression levels at higher pH had a strong association with synaptic functions [40, 49]. The current findings are consistent with those previous results. In fact, synapse loss has been observed in AD both in humans and animal models of the disease [69] and it is known to be strongly correlated with cognitive decline [70, 71]. On the other hand, neural hyperexcitation‐associated genes in AD model mice were enriched in inflammatory responses. Neuroinflammation has been implicated in AD mouse models [72]. In a previous study involving 24‐h electroencephalographic recordings of AD patients and healthy controls, subclinical epileptiform discharges were significantly more frequently recorded in AD patients compared to healthy elderly individuals [73]. It is known that various inflammatory markers, such as interleukins (ILs) and tumor necrosis factor‐α (TNF‐α), are elevated in the plasma of AD patients compared to control groups [74, 75]. Among these, TNF‐α, IL‐1β levels and its receptor IL‐1R1 in human serum have been suggested to correlate with the severity of epilepsy [76]. These findings suggest that both AD and epilepsy may share common inflammatory responses in the brain [77, 78].

Regarding the potential relationship between immaturity, decreased pH, and neural hyperexcitation in AD, the following hypotheses can be proposed based on previous studies using animal and cell models. Deposition of Aβ/tau, neuroinflammation, and neural hyperexcitation are known to induce each other [79, 80, 81], although the sequence in which these processes occur remains unclear. Neural hyperexcitation has been reported to promote increased glycolysis, leading to elevated lactate production and a subsequent decrease in pH [82]. Furthermore, neural hyperexcitation is suggested to induce the immaturity phenotype in mice [14, 24, 83] and humans [49]. In mouse exhibiting the iDG phenotype, reductions in synapse‐related molecules have been observed [19, 84], along with morphological [85] and electrophysiological characteristics [13, 86] indicative of immaturity. Hyperexcitation, decreased pH, and immaturity have also been suggested in certain brain regions of patients with AD [36, 87, 88]. Further studies are needed to elucidate the exact mechanisms underlying the causal relationships among these three phenomena in the brains of patients with AD.

There are several limitations to consider when interpreting the findings of this study. First, we did not perform analyses to account for potential confounding factors.

Potential confounding factors that could contribute to variability among datasets include differences in sex, sampling hippocampal sub‐regions analyzed, and analysis platforms (microarray or RNA‐sequencing). Since most of the datasets used in this study are derived from female samples and whole hippocampus, we believe that sex and regions are unlikely to significantly influence the results (Table S1). Furthermore, we consider it unlikely that the observed variability among datasets is attributed to differences in analysis platforms. Nonetheless, we cannot exclude the possibility that unknown factors may confound our findings. Second, we did not conduct analyses considering cell type specificity. It is known that, in the AD model mice analyzed in this study, not only neuronal alterations but also changes in other cell types, such as activation of microglia and astrocytes, as well as abnormalities in oligodendrocytes, were observed [66, 89, 90, 91]. However, the extent to which such cell type‐specific changes might influence the observed variability among datasets remains unaddressed.

In conclusion, gene expression patterns related to maturity, pH changes, and neural hyperexcitation may reflect the progression of AD. Gene expression patterns of these signature may serve as valuable markers for assessing the severity of brain pathology in AD model mice. They may also provide new markers into AD pathology at the cellular and molecular levels, offering clues for the development of novel prevention and treatment strategies based on these insights.

## Author Contributions

H.H. and T.M. developed the study concept. All authors collected, analyzed the data, wrote the paper, and approved the final manuscript.

## Ethics Statement

The authors have nothing to report.

## Consent

The authors have nothing to report.

## Conflicts of Interest

The authors declare no conflicts of interest. Tsuyoshi Miyakawa is the Editor‐in‐Chief of Neuropsychopharmacology Reports and a co‐author of this article. They were excluded from editorial decision‐making related to the acceptance and publication of this article. Editorial decision‐making was handled independently by Editor‐in‐Chief, Tsuyoshi Miyakawa, to minimize bias.

## Supporting information


Table S1.

## Data Availability

The datasets presented in this study can be found in BaseSpace (https://basespace.illumina.com/) and the Gene Expression Omnibus (https://www.ncbi.nlm.nih.gov/geo/).
